# Supplementary material for: Photothermal Therapy Combined with Chemotherapy and Anti-Inflammation Therapy Weakens the Immunosuppression of Cervical Cancer
Source: Pharmaceuticals (Basel). 2025 Nov 1;18(11):1657. doi: 10.3390/ph18111657 (PMC12654961; doi:10.3390/ph18111657)
Supplement: Supplementary file 1 [file pharmaceuticals-18-01657-s001.zip › pharmaceuticals-3933728-supplementary.pdf]

Supplementary figures

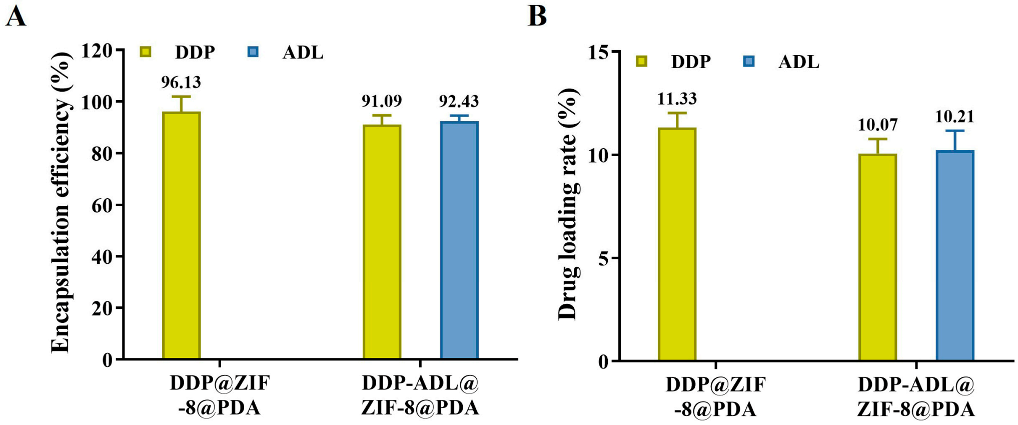

Figure S1. Drug encapsulation efficiency and loading efficiency testing. **A.** DDP and ADL encapsulation efficiency test; **B.** DDP and ADL loading efficiency testing.

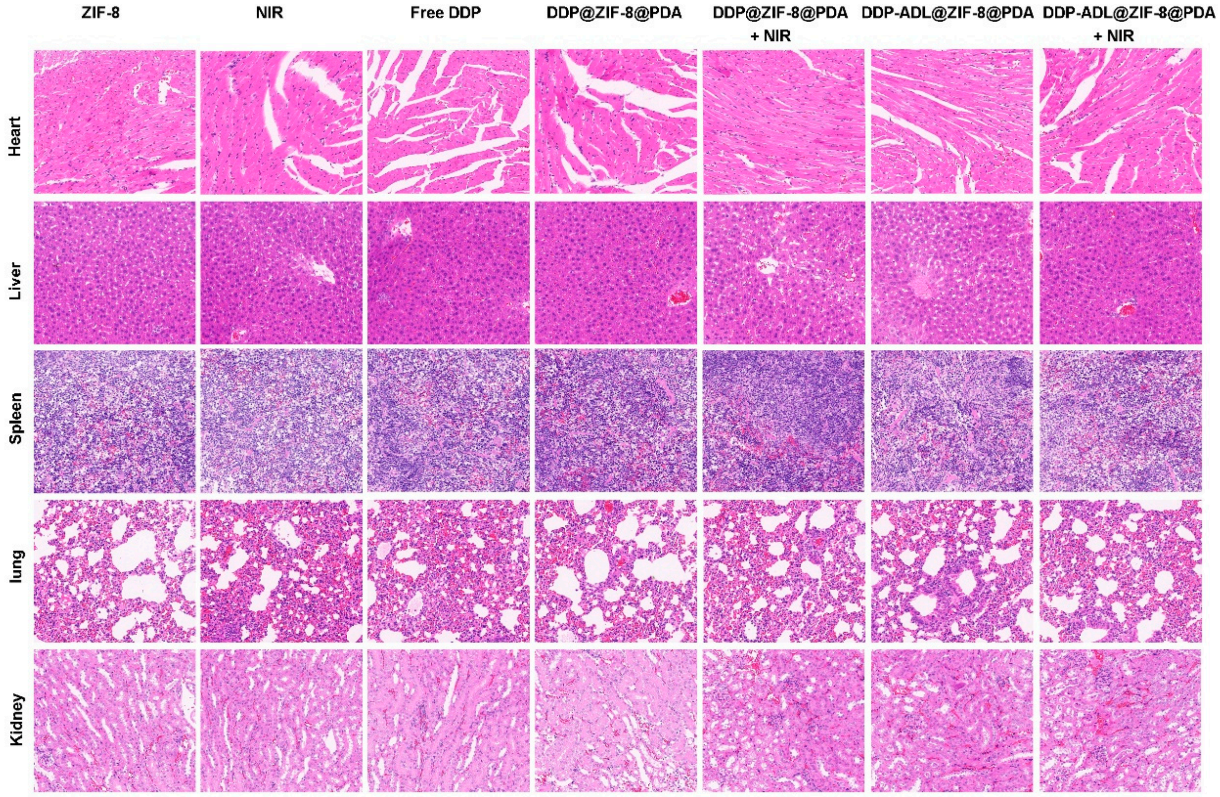

Figure S2. Major organs (heart, liver, spleen, lung, and kidney) from the tumor bearing mice in different treatment groups with H&E staining after different treatment.
